# Digitized Prenatal Newsletter: Impact on Obstetric Patient Satisfaction and Loyalty

**DOI:** 10.3390/ijerph19052773

**Published:** 2022-02-27

**Authors:** María Caballero-Galilea, Esther Martínez-Miguel, Juan Carlos Fernández Gonzalo, Ricardo Saiz de la Cuesta Abbad, Margarita Rubio Alonso

**Affiliations:** 1 Department of Nursing and Nutrition, Faculty of Biomedical and Health Sciences, Universidad Europea de Madrid, 28670 Madrid, Spain; maria.caballero@universidadeuropea.es (M.C.-G.); juancarlos.fernandez@universidadeuropea.es (J.C.F.G.); 2 Departament of Gynecology and Obstetrics, Hospital Universitario Quirónsalud Madrid, 28223 Madrid, Spain; ricardo.sainzdelacuesta@outlook.com; 3 Department of Medicine, Faculty of Biomedical and Health Sciences, Universidad Europea de Madrid, 28670 Madrid, Spain; margarita.rubio@universidadeuropea.es

**Keywords:** prenatal care, maternal health services, gynecology, obstetrics, information needs, satisfaction, loyalty

## Abstract

The high demand for health information from pregnant women has encouraged the creation of an informative program through a weekly digital newsletter. The objective of this study is to evaluate its quality as a digital communication medium, in terms of satisfaction and loyalty to the pregnancy follow-up and delivery service. A cross-sectional, prospective study was carried out, surveying 179 patients by means of an online self-referral questionnaire including variables related to humanization, information needs, perceived accompaniment and satisfaction, as well as factors related to its influence on their decision to remain loyal to the center. A total of 81.2% of the participants showed high levels of satisfaction with the program. Satisfaction among nulliparous patients was significantly lower in several aspects. The resolution of doubts and the perception of peace of mind following the information received was positive for 54.8%. Of the patients in the program, 88.8% finally remained at the center, showing a strong influence of the program on their decision (mean value 75 on 1 to 100 scale). A weekly digital newsletter with specific information reduced the demand for information from pregnant women, generating high levels of satisfaction and positively influencing the decision to remain loyal to the Center.

## 1. Introduction

The decrease in the birth rate will have general economic repercussions underlying the inversion of the population pyramid, including an imbalance in the pension system and the healthcare system, while at the same time reducing one of the main groups of users of private healthcare centers, pregnant women. This decline has led to the implementation of improvements in their care processes, as a result of an emerging competition among private centers to ensure the loyalty of pregnant patients. At present, the challenge is to apply relationship marketing strategies to understand the needs of users and prioritize them within the objectives of the institution [1] improving the patient experience to improve their commitment to the center [2,3], especially within the private health [2,4]. In a previous study by the authors of this work, the results showed that transmitting and adequately communicating information related to the pregnancy process is a fundamental factor in achieving the loyalty of pregnant women [5].

It is known that the information needs of pregnant women are very high from very early stages of pregnancy [6], thus NICE (National Institute for Health and Care Excellence) recommends that the first visit should take place before week 10 in order to respond to this demand for information [7]. Providing information encourages patients to understand, remember and adhere to recommendations and/or treatments, with a positive impact on health outcomes [8,9], improving eating and exercise habits which are associated with a more positive birth experience [9,10]. Pregnant women positively value expert professional advice and express the desire to obtain wider and faster access to information through digital technologies [11]. Some studies show, however, that increased use of digital media can have a potentially negative effect on the psychological well-being of pregnant women. In this sense, it is crucial that they have access to quality evidence-based information regardless of the route chosen to obtain it, through in-person or digital interactions, also needing in some cases psychological support [12]. Health providers must recognize these needs and include in their service planning policies ways to satisfy them, providing the necessary support to this group [11].

Healthcare management models must adapt to the use of web tools that improve overall communication, especially between inter-hospital processes [4]. The World Health Organization (WHO) considers that digital health is key to transmitting content to a particular sector of the population, depending on their general health status or sociodemographic profile. This dissemination includes a wide variety of content on health promotion, information on reference healthcare centers, etc. [13], which is an opportunity to improve people’s commitment to their health and their relationship with the center. Digitalization promotes user loyalty to the center, especially in those processes that require continuous monitoring [3,14,15,16].

Health education programs should provide selective information tailored to the target population [17,18,19] chronologically, from the first preconception visit to the postpartum period, and should be aligned with both office follow-up programs and routine diagnostic tests [7,19]. Digitization allows supplementary sources of information to resolve doubts related to health and that of families [20] and the knowledge with which the patient arrives at the consultation is key, since when it is adequate, it allows it to be optimized [21,22], in addition to reducing the number of face-to-face visits, without impacting the quality of care, nor the satisfaction of patients with the center [23]. During the current pandemic situation and mediated by the powerful acceleration of digital transformation, many pregnant women have sought to satisfy their need for information through social networks. These have positioned themselves as a source of health information, even calming the anxiety produced by the experience of lockdown (confinement) and the absence of direct medical assistance, which has been highly valued by women [24,25].

User loyalty with the health center and/or service is closely linked to patient satisfaction, showing especially positive results in cases of reproductive health [15,16]. At present, we have not found any studies analyzing the relationship between maternity programs and user loyalty [26].

Following the observations and data obtained in the previous study mentioned above [5], the following questions arise: (1) Can the experience of pregnant patients be improved with the implementation of a digital information program adapted to pregnancy age by sending newsletters? (2) How does this program influence their satisfaction and loyalty?

The objective of this study is to evaluate the quality of a newsletter as a digital communication medium aimed at pregnant women in terms of satisfaction and loyalty to the pregnancy follow-up and delivery service.

## 2. Materials and Methods

**Figure A1:**
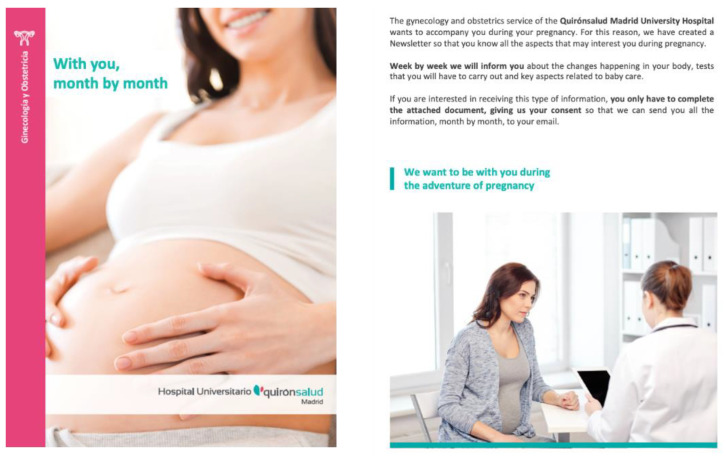
Flyer informing about the digital Newsletter ‘Contigo mes a mes’ program (prepared internally and by the HUQSM marketing department).

**Figure A2:**
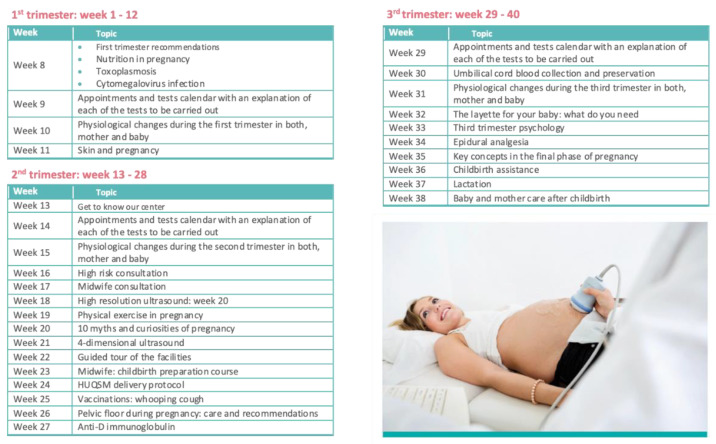
Flyer informing about the contents of the ‘Contigo mes a mes’ program (prepared internally and by the HUQSM marketing department).

Participants were recruited at the Hospital Universitario Quirónsalud Madrid (HUQSM). The study was approved by the Clinical Research Ethics Committee of the Fundación Jiménez Díaz (no. 21/20169). This hospital offers pregnant women a weekly newsletter called ‘Contigo mes a mes’ (‘With you, month by month’) with information and advice between the 8th and 38th week of pregnancy (Appendix A and Appendix B). The contents were developed by the interdisciplinary obstetrics team based on the lack of information on the center’s website and the information needs most frequently requested by patients in consultation (Figure A1 and Figure A2).

A cross-sectional, prospective study was carried out to determine the satisfaction and loyalty of the pregnant women with the health center after the implementation of the program. Data collection was carried out between July 2019 and November 2020, by means of an anonymized questionnaire and consecutive convenience sampling. The patients received the informed consent, the information sheet of the study and the questionnaire along with the latest Newsletter. The exclusion criteria were considered to be pregnancy termination and voluntary withdrawal from the program.

For the development of the questionnaire, the Delphi methodology used in the previous study at the same center [5] was replicated, seeking an integral and multidisciplinary vision through the involvement of different health professionals who participated in the process. The survey had 22 main items and an open space for free qualitative contributions (Appendix C).

The questionnaire variables included sociodemographic aspects, the obstetric profile, and dichotomous questions with ‘Yes’ and ‘No’ response options, with ‘Maybe’ as an alternative ‘No’. Trichotomous and multiple-choice variables were used in a variety of shades of meaning with respect to what was being asked. The open-ended questions closed the questionnaire in order to offer the freedom to provide more information regarding the experience. The Likert scale was used as an instrument to measure satisfaction and experience, where 1 was considered very dissatisfied and 5 very satisfied. Subsequently, in the analysis, indicators of dissatisfaction were considered to be those between 1 and 3, and those between 4 and 5 were considered to be indicators of satisfaction.

Continuous study variables were described as the median and interquartile range (IQR) or mean and standard deviation (SD) according to their normal behavior. Qualitative variables were presented as absolute frequencies and percentages. To make comparisons between the different groups, the chi-square test or Fisher’s exact test was used for qualitative variables. The comparison of quantitative variables was performed using the *t*-student test for independent samples when two groups were compared; in the case of more, the test used was ANOVA. If any variable did not follow a normal distribution, the corresponding nonparametric tests were used. Statistical significance was considered when the *p*-value was less than the alpha error, which for this study was considered to be 5%.

## 3. Results

A total of 844 pregnant women were registered for the newsletter, between February 2019 and November 2020. The questionnaire was sent to a total of 441 women who had completed pregnancy, 66.4% (293/441) were registered for the newsletter during their first trimester of pregnancy. 30.15% in the second quarter (133/441) and 3.4% (15/441) in the third quarter. The response rate was 65.3% (288/441), 91 questionnaires were excluded from the analysis because they were incomplete, and 197 were finally analyzed.

Participants (*n* = 197) ranging in age from 22 to 48 years with a median of 36 (RIC: 33–39). A total of 43.7% (*n* = 86) lived in Madrid, compared to 51.3% (*n* = 101) who came from outlying towns near the health center. A total of 94.9% (*n* = 187) were users of the center with an insurance company policy. Sixty-eight percent (133/197) of the women were nulliparous and 26.4% (52/197) were diagnosed with high-risk pregnancy. A detailed description of the sociodemographic characteristics of the sample can be found in Table 1.

Of the patients, 32.5% (64/197) had given birth previously, 59% (38/64) gave birth at the Center (HUQSM), while the rest (12/64; 19%) gave birth at another private or public center (14/64; 22%).

Participants were registered for the newsletter between 5 and 35 weeks of pregnancy (median = 12 weeks). A total of 52.3% (103/197) of the registrations took place in the first quarter, while 40.1% (79/179) took place in the second quarter. Furthermore, 80.2% (158/197) of the patients learned about the program during the in-person consultation at the Center, 17.3% (34/197) through the HUQSM website, and 2% (4/179) through the recommendation of family or friends. The median number of emails received per patient was 23 (minimum 4 and maximum 32). Regarding satisfaction with the information received through the newsletter, 56.9% of the participants (112/197) would have preferred to receive more information, 36.5% (13/197) answered ‘Maybe’ and 6.6% (13/197) answered ‘No’. A total of 59.4% (117/197) considered that all their doubts had been resolved and did not feel the need to ask their gynecologist in consultation.

**Figure 1:**
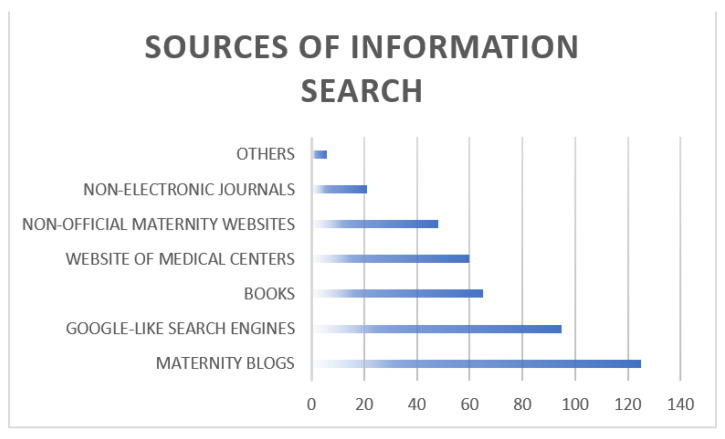
Sources of information search: preferred Internet search sites for pregnant women.

Pregnant women were asked to list their preferred Internet search sites among 7 alternatives (Figure 1). The majority pointed to maternity blogs (63.5%), and search engines (48.2%). Only 16.2% made additional queries using the center’s email address provided for resolving doubts.

A total of 93.9% (185/197) preferred the direct sending of information in newsletter format compared to 6.1% (12/197) who preferred to consult it by means of an autonomous search. 73.1% (144/179) shared information with their partners.

The results obtained by means of a Likert scale were used to evaluate satisfaction, showing that 81.2% (160/197) felt ‘satisfied’ with the program (4–5 on the Likert scale). In addition, 75.6% (149/179) considered the contents to be appropriate for the week of pregnancy, and 75.6% (149/197) felt that they were more accompanied during the process.

No association was found between the age of the woman and satisfaction with the information received (*p* = 0.054), nor satisfaction according to the quarter of registration for the newsletter (*p* = 0.267). We did observe a slight decrease in satisfaction levels if the patient was included in the program late (85.40% satisfaction if inclusion was during the first trimester, 77.2% in the second, and 73.3% during the third; *p* = 0.267).

In the questions on satisfaction with the thematic blocks of the newsletter (Table 2), the highest percentages of satisfaction correspond to: ‘Changes in pregnancy’ and ‘Tests to be performed during pregnancy’ (78.2% and 77.7% ‘satisfied’ or ‘very satisfied’, respectively). There was less satisfaction with aspects related to childbirth and postpartum compared to antepartum stages.

Satisfaction was significantly lower among nulliparous patients compared to secundiparous and multiparous patients with respect to information received about tests to be performed during pregnancy (73.2% vs. 88.1%; *p* = 0.021) and about facilities (51.4% vs. 67.8%; *p* = 0.034). There was no difference in satisfaction with the program according to whether the outcome was delivery or cesarean section (*p* = 0.711). 

The resolution of doubts and the perception of peace of mind following the information received were positive for 54.8% (108/197), while 34% (67/197) reported ‘maybe’ and 11.2% (22/197) did not report feeling more at ease. A total of 55.8% (110/197) said that they were calmer at delivery thanks to the information received, 32.5% (64/197) were not sure (‘maybe’) and 11.7% (23/197) said they were not calmer. Perceived calmness was not associated with the outcome of delivery (vaginal or cesarean) (*p* = 0.51). Of those who had a cesarean section, 57.4% (31/54) responded that the information had given them more peace of mind compared to 56.2% (68/121) of those who had a vaginal delivery.

Of the patients in the program, 88.8% (175/197) finally remained at the center. Of the 11.2% who did not remain at the center, 5 were multiparous and 17 were nulliparous. All multiparous women who had previously had children in other private centers showed intention to finish the process in the center (100%; 11/11); of those who had their children in public centers, 55.6% (5/9) and of those who had them in HUQSM, 84.6% (33/39) (*p* = 0.003). Of the total number of patients, 14.7% (29/197) underwent parallel check-ups with the public health system and 9.1% (18/197) sought a second opinion.

**Figure 2:**
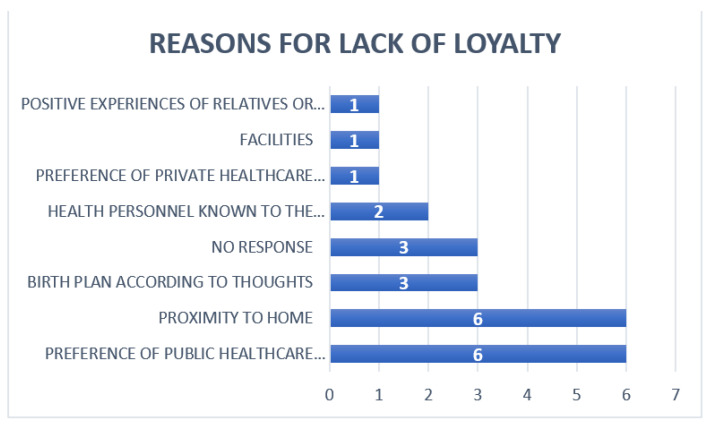
Reasons why they do not gain loyalty to the pregnancy follow-up and delivery service.

As a reason for choosing the center for the process, ‘previous experiences of family and friends’ was reported by 28.4% (56/197), ‘birth plan according to expectations’ by 21.8% (43/197), and ‘proximity to home’ by 17.8% (35/197). The rest of the reasons referred to the preference of private healthcare over public healthcare 10.20% (20/197) and 5.58% (11/197), respectively, or healthcare personnel known in the center 10.10% (20/197). The reasons for the patients who did not gain loyalty are equally diverse, as can be seen in Figure 2.

Before the end of the pregnancy process, the questionnaire was sent to the pregnant women and they were asked about their intention to remain loyal to the Center. A total of 84.8% (167/197) believed that they would stay with the center, while 11.7% would stay at another center (23/197), and 3.6% (7/197) had not yet decided. Of the multiparous women who gave birth at HUQSM, 97.4% (37/38) chose to give birth at HUQSM again. Of those who gave birth in another private center, 83.3% (10/12) intended to give birth at the HUQSM Center. Among those who gave birth in centers of the public health system, 71.4% (10/14) intended to give birth in HUQSM and 14.3% (2/14) in the public health system. Of the total, 3.12% (2/64) remained hesitant about where to give birth.

**Figure 3:**
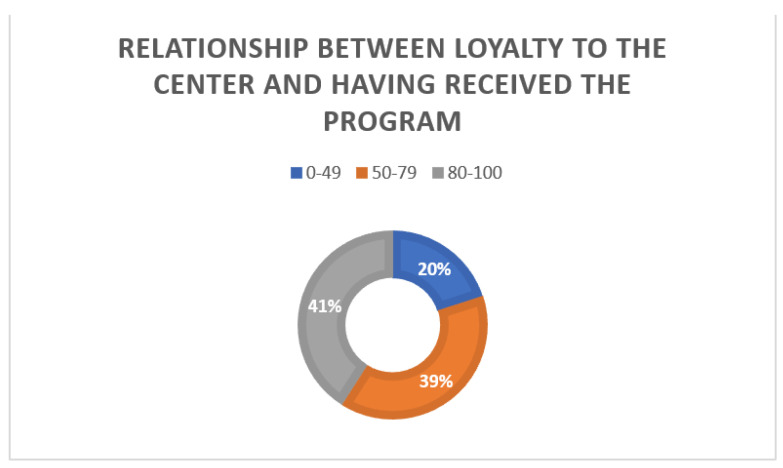
Influence of the program on the decision to become loyal to the center on a scale of 0 to 100.

On a scale of 1 to 100, they rated the degree to which the information received influenced their decision to stay with the center, with the mean value being 75. Moreover, 40.6% (*n* = 80) of the surveys obtained values between 80–100 (Figure 3).

## 4. Discussion

In this study, we show how the use of a digital information program through the sending of a newsletter designed for pregnant women achieved good results in terms of satisfaction and loyalty with the center.

Among the different aspects that could be related to good results in terms of satisfaction and loyalty to a pregnancy follow-up and delivery service is the empowerment of women in the care of their health. There is a high demand for information on the pregnancy process from very early pregnancy stages, motivated by physical, psychological, and emotional changes, as well as by the socio-family and work role transformation that accompanies motherhood [6,27,28]. To meet this demand, professionals must offer continuous support and information, adapted to the needs of pregnant women and available in real time [16,29].

In recent years, digitization is responding to the demands for information and assistance through mobile health applications (mHealth), being characteristic in maternal and fetal health, searches related to the physical changes of the mother, child development or diagnostic tests during pregnancy, ranging from preconception to parenting and childcare [15,30]. Evaluation of the effectiveness of these health apps shows increases in women’s satisfaction with the medical care received and increased adherence to services and health recommendations during pregnancy, which is consistent with the satisfaction shown in the results of our study [15].

Receiving personalized notifications with information from applications or websites provides women with peace of mind and can prevent disorders such as anxiety and depression [31], which are often manifestations of the uncertainty and lack of knowledge typical of this period [6,27,28]. In our study, the information provided in each newsletter mailing generated high levels of satisfaction for the item ‘perceived accompaniment’, which could reduce or avoid the feelings described.

Even when pregnancy is not yet physically manifested, women demand information. It is about being able to mentally represent the process, especially the physical changes that occur in embryonic and fetal development, which is directly related to the adoption of healthy behaviors [27,32]. In this sense, the lack of previous experience of the pregnant woman makes a difference in relation to the feelings of uncertainty and the need to control the process, which would explain the lower satisfaction of nulliparous women with the program.

Educating and motivating are key objectives to achieve changes towards healthier behaviors and greater involvement and/or participation in one’s own healthcare [9,17]. Health education programs designed for pregnant women should be personalized to achieve adherence and optimal outcomes, tailored to each stage of pregnancy, with up-to-date information provided in a timely and empathetic manner [14,27]. The most commonly used websites and apps are those that track the stages of pregnancy week by week, describing fetal development and body changes, which helps them to sequentially conceptualize the changes taking place in their body [21,32]. This is consistent with the results of this study, as the weekly adaptation of newsletter content is rated very positively.

Regarding the way of accessing information, there is a clear preference for weekly reception of content as opposed to active access or search. Women show a preference for the fact that the information comes from healthcare professionals [27], that it is of quality, questioning the reliability of web sources and the security of personal data collected by Apps [21,30]. Pregnant women express reluctance to use shared blogs due to the lack of privacy, security, and reliable information, so dubious information sources are not perceived as the most appropriate for health education [33]. This is, however, a controversial aspect, as some studies warn that the wide availability of apps and websites to obtain an immediate response to their information needs may reduce the demand for attention to health professionals, in favor of non-scientific sources [30,31]. Sending a newsletter from the hospital ensures evidence-based information and guarantees privacy in every communication with the pregnant woman. The weekly periodicity in a vital process such as gestation allows personalizing and adjusting the information to individual needs.

Involving the couple in the information-seeking process is key to improving the bond between the couple and the developing fetus [32,34,35,36]. In our study, participants reported sharing information with their partners in a high percentage of cases.

The use of digital communication directed to the patient through various channels such as blogs, articles on social media is an effective form of communication after an interaction or user experience with the health center [37]. Expert professional through digital technologies is highly valued as it offers quick, easy, and secure access to information [11]. This is related not only to the implementation of the program but also to the information search sources used by the patients to resolve their doubts, in maternity blogs in 63.5% of the cases. In the study, more than half of the participants considered that all their doubts were resolved through the newsletter, which could lead to more targeted obstetric consultations.

Satisfaction is conceptualized as the comparative relationship between the perception of a service and the expectations regarding it, directly impacting customer loyalty [38,39,40,41,42]. With respect to loyalty or intention to return to a healthcare center, satisfaction is strategic, since when a user has a positive relationship with the organization, their loyalty and willingness to recommend the organization to other potential users increases [43]. In our study, the rate of loyalty to the center in patients who received the program is high, and this figure is even higher in multiparous women with previous experience at our center. 

Aspects such as physical and human environment, communication and responsiveness, privacy, and security are positively related to patient loyalty, which is mediated by patient satisfaction [44]. The Newsletter was designed to respond to the information needs of pregnant women and to meet the communication expectations during the inter-visit periods during follow-up visits. The women surveyed rated positively the influence of the ‘Contigo mes a mes’ program on their decision to stay with the center.

Women say that the information provided by the newsletter helps them feel more prepared and more relaxed at the time of delivery, but satisfaction decreases as the time of delivery, postpartum, and breastfeeding approaches. Several studies show that the demand for postpartum information is equal to or greater than in the early stages of pregnancy in nulliparous pregnant women, despite childbirth preparation courses. The psycho-emotional and preparatory aspect is crucial and there is a great lack of information regarding these stages [19], a fact that increases in the postpartum, breastfeeding, and upbringing periods [6,45,46,47].

Healthcare providers, facilities, and the professionals who make them up have the opportunity to capitalize on the high rate of web-based information seeking through patient-facing digital communication initiatives [31]. Responding to individualized information needs [48] has a positive impact on patient satisfaction with the service and care received, and this, in turn, modulates positive loyalty behaviors [15].

The study was limited by the fact that patients were selected from a single center, limiting its external validity. The use of the survey as a data collection tool is considered as information bias, which, being a self-reported method, does not allow for independent verification of the data. Finally, the cross-sectional nature of the study does not allow us to establish causal associations between the variables.

Given that there is a gap between the supply of outreach channels and applications aimed at pregnancy and those available for the postpartum and early parenting period, we propose as a future line of research the study of information demands in relation to the transition between pregnancy and postpartum, including general aspects of parenting. These studies could serve as a starting point for designing and implementing patient-focused communication programs tailored to these needs, with the aim of improving maternal and neonatal health and client satisfaction and loyalty.

## 5. Conclusions

The design and implementation of the ‘Contigo mes a mes’ program, a digital newsletter with specific information for each week of pregnancy, reduced the demand for information from pregnant women during the follow-up of their pregnancy. 

As a means of digital communication addressed to the patient, the information received through the newsletter generates high levels of satisfaction and influences the decision of loyalty to the Center for the delivery care.

## Figures and Tables

**Table 1:** Sociodemographic variables.

Variables	*n* = 197	%
Age		
20–29	10	5.10%
30–35	87	44.2%
36–40	64	32.5%
>40	36	18.3%
Population of residence		
Madrid City	86	43.7%
Other towns in the Autonomous Community	101	51.3%
Other Autonomous Community	10	5.1%
Educational level		
Basic training baccalaureate	6	3.05%
University or graduate studies	191	97.0%
Obstetric pattern		
Nulliparous	133	67.5%
Secundiparous	55	27.9%
Multiparous (2 children)	9	4.6%

Description of the sociodemographic characteristics of the sample.

**Table 2:** Themes of the newsletter and satisfaction according to obstetrical pattern.

Theme of the Newsletter	Satisfaction	Total		Secundiparous/Multiparous		Nulliparous		*p*
		*n*	%	*n*	%	*n*	%	
Tests to be performed during pregnancy	NEUT_OR_UNSAT	44	22.30%	7	11.90%	37	26.80%	0.021
	SAT_OR_VERYSAT	153	77.70%	52	88.10%	101	73.20%	
Pregnancy healthcare	NEUT_OR_UNSAT	49	24.90%	11	18.60%	38	27.50%	0.186
	SAT_OR_VERYSAT	148	75.10%	48	81.40%	100	72.50%	
Pregnancy changes	NEUT_OR_UNSAT	43	21.80%	9	15.30%	34	24.60%	0.144
	SAT_OR_VERYSAT	154	78.20%	50	84.70%	104	75.40%	
Childbirth: symptoms, measures, labor process	NEUT_OR_UNSAT	56	28.40%	13	22.00%	43	31.20%	0.193
	SAT_OR_VERYSAT	141	71.60%	46	78.00%	95	68.80%	
Changes during pregnancy	NEUT_OR_UNSAT	73	37.10%	19	32.20%	54	39.10%	0.356
	SAT_OR_VERYSAT	124	62.90%	40	67.80%	84	60.90%	
Anesthesia	NEUT_OR_UNSAT	78	39.60%	22	37.30%	56	40.60%	0.357
	SAT_OR_VERYSAT	119	60.40%	37	62.70%	82	59.40%	
Breastfeeding	NEUT_OR_UNSAT	86	43.70%	19	32.20%	67	48.60%	0.665
	SAT_OR_VERYSAT	111	56.30%	40	67.80%	71	51.40%	
Facilities (neonatal and adult ICU)	NEUT_OR_UNSAT	86	43.70%	19	32.20%	67	48.60%	0.034
	SAT_OR_VERYSAT	111	56.30%	40	67.80%	71	51.40%	
Special services (High-risk unit and prenatal diagnosis)	NEUT_OR_UNSAT	89	45.20%	21	35.60%	68	49.30%	0.077
	SAT_OR_VERYSAT	108	54.80%	38	64.40%	70	50.70%	

Comparison of satisfaction with the different topics received with the program and its contents according to obstetrical pattern (NEUT_OR_UNSAT: neutral or unsatisfied; SAT_OR_VERYSAT: satisfied or very satisfied).

**Figure A3:**
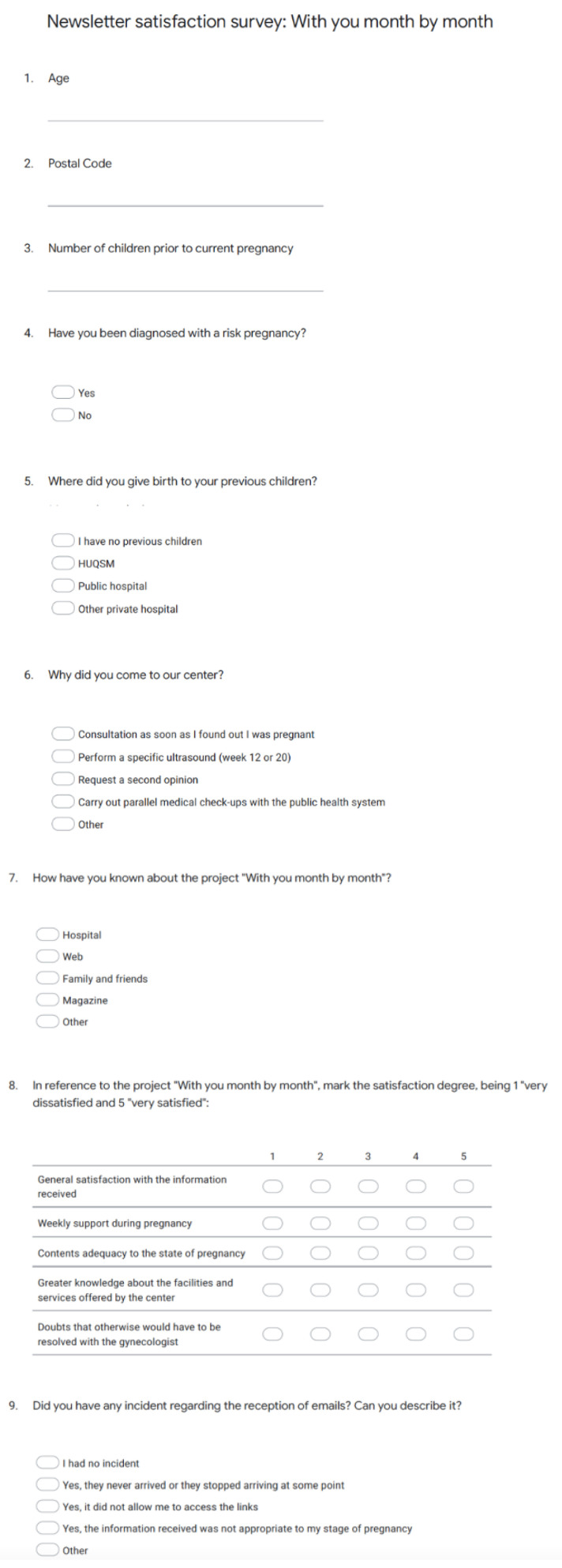

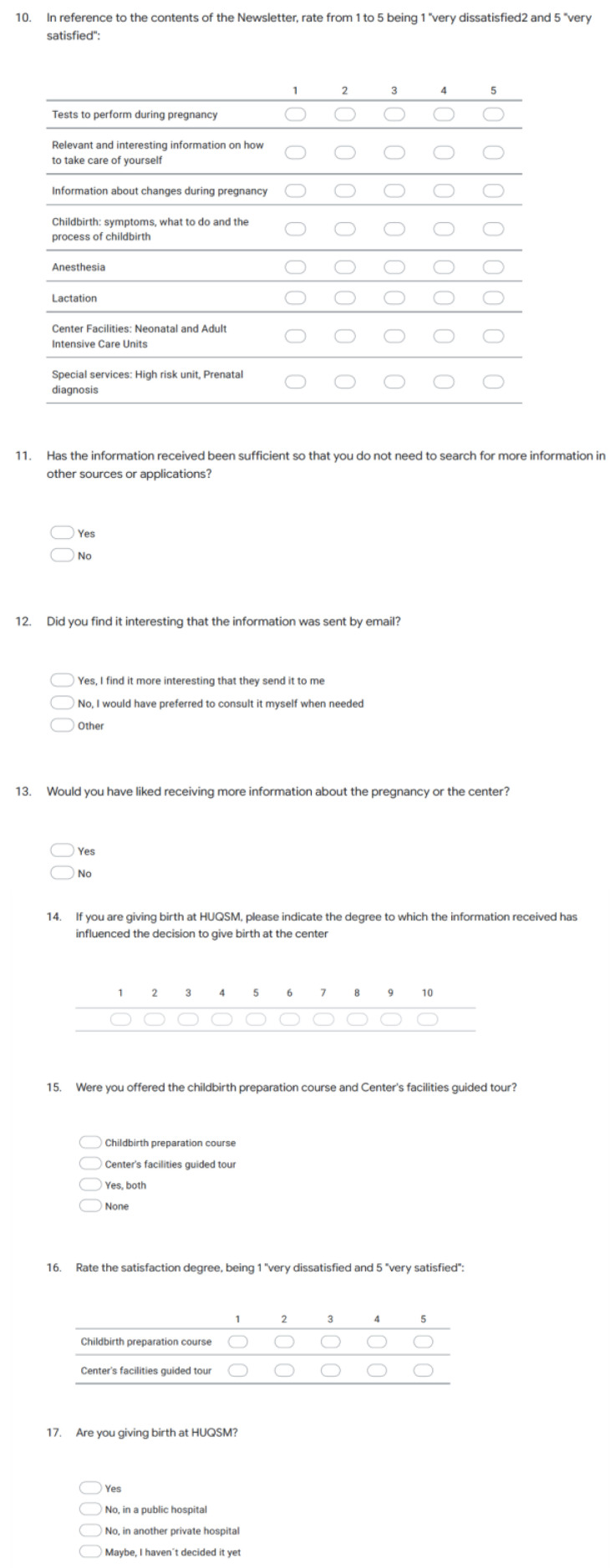

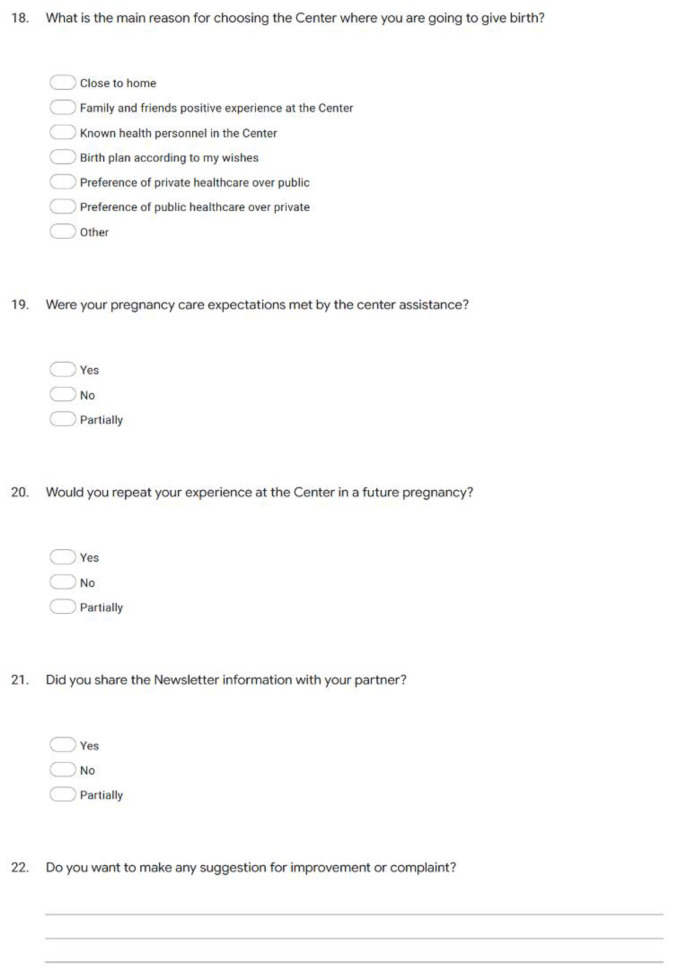
Survey.

## Data Availability

Data available on request due to restrictions related to the General Data Protection Regulation 2016/679. The data presented in this study are available on request from the corresponding author.

## Appendix A

*Presentation of the program ‘Contigo mes a mes’ (‘With you, month by month’).*

## Appendix B

*Themes of the program ‘Contigo mes a mes’ (‘With you, month by month’).*

## Appendix C

*Newsletter survey.*
